# A clustering method for graphical handwriting components and statistical writership analysis

**DOI:** 10.1002/sam.11488

**Published:** 2020-11-24

**Authors:** Amy M. Crawford, Nicholas S. Berry, Alicia L. Carriquiry

**Affiliations:** ^1^ Department of Statistics Iowa State University Ames Iowa USA; ^2^ Berry Consultants Austin Texas USA

**Keywords:** Bayesian, clustering, forensic statistics, handwriting analysis, hierarchical modeling

## Abstract

Handwritten documents can be characterized by their content or by the shape of the written characters. We focus on the problem of comparing a person's handwriting to a document of unknown provenance using the shape of the writing, as is done in forensic applications. To do so, we first propose a method for processing scanned handwritten documents to decompose the writing into small graphical structures, often corresponding to letters. We then introduce a measure of distance between two such structures that is inspired by the graph edit distance, and a measure of center for a collection of the graphs. These measurements are the basis for an outlier tolerant *K*‐means algorithm to cluster the graphs based on structural attributes, thus creating a template for sorting new documents. Finally, we present a Bayesian hierarchical model to capture the propensity of a writer for producing graphs that are assigned to certain clusters. We illustrate the methods using documents from the Computer Vision Lab dataset. We show results of the identification task under the cluster assignments and compare to the same modeling, but with a less flexible grouping method that is not tolerant of incidental strokes or outliers.

## 1 INTRODUCTION

Many disciplines rely on the ability to parse, process, and analyze handwritten text. Examples include automatic mail sorting using zip codes, determination of authorship of old manuscripts, and forensic examination of handwritten documents. In recent years there has been a shift towards the automation of some of the text parsing and processing steps. Briefly, automatic handwriting processing is the task of converting an image of handwriting into usable data that can then be input into various statistical approaches. The data extracted for analysis depend on the type of analysis and specific application. When done algorithmically, handwriting analysis usually falls into one of two categories. One common analysis objective is to recognize the characters written on a page, as in the case of mail sorting. To carry out this task, organizations that receive and deliver mail use algorithms that can recognize zip codes to sort correspondence. A different type of analysis is a determination of the person who may have written the document, which is called writer identification. For writer identification, the goal does not concern investigating *what* is written, but the *way* in which it is written.

Within the writer identification framework, we distinguish between authorship and writership. Authorship analyses often use word choice or punctuation in a document (e.g., Rosen‐Zvi et al. [16] or Seroussi et al. [18]) and can involve a mix of the two objectives described above. An early application of authorship analysis was carried out by [13] to identify the authors of the unsigned Federalist Papers. Writership identification however, is limited to an analysis of the *shapes* that a writer emits via their practiced writing style. We focus on this latter sub‐field of handwriting analysis.

One large and active use of writership identification is in forensic practice. Such analyses can be used to determine the source of a piece of handwritten evidence, for example, a bank robbery note or a bomb threat. Traditionally, handwriting analysis of this nature is done by trained forensic practitioners who rely on their own training and experience to carry out a subjective evaluation of the writing samples. In practice, an examiner reaches a conclusion regarding writership using a decision scale such as the 9‐point scale of ASTM Standard E1658‐08 [1]. The scale includes terms like “identification,” “probable,” and “elimination,” that are used when making a comparison between a document from an unknown writer and one from a known writer. The ASTM standard has since been withdrawn, but similar best practices continue to be in use.

The work we discuss in this paper addresses the problems of automating and quantifying portions of the forensic handwriting examination process. In particular, we pursue the following goals:

1. Develop a set of rules (and the software to implement them) to parse a handwritten document into graphical structures.
2. Propose a dynamic and flexible method to group those graphs into clusters with similar characteristics, generating a clustering template to be used as a feature extraction tool for future handwriting samples.
3. Use the clustering template to classify graphs from new handwritten documents. Use the classifications as a primary feature in a statistical model, to explore how a writer's propensity for creating graphs that fall into each cluster can be used in a writership analysis.

We use scanned handwritten documents from a variety of writers in the Computer Vision Lab (CVL) database [8] to meet our goals.

In this writership analysis framework, there are two stages of feature extraction that occur. First, we construct a sequence of disjoint graphical structures to represent the ink displayed on scanned images of documents. In the second stage, each graph in a document is compared to a clustering template and is assigned to one of the clusters based on similarity to template components. To generate the clustering template, which acts as a feature extraction tool, we develop our own distance and cluster center metrics (see Section 3.2).

A question of interest is whether writers can be distinguished by the proportion of the graphs extracted from their writing that fall into each of the *k* clusters in the template. To address this question, we use the observed cluster frequencies in a document by a writer as the response variable in a hierarchical model to estimate the posterior probability of writership for each writer in a closed set. We choose a multinomial distribution with *k* categories, for *k* the number of clusters, to model the cluster frequencies. The hierarchical model can then be used to estimate the multinomial parameters for each writer, and compute posterior predictive probabilities of writership. To test the predictive ability of the model, we hold one document back from each writer in the closed set; this held‐back document is not used to create the cluster templates or to estimate the parameters of the model.

We are not the first to work on this problem. Our work is inspired by methodology proposed earlier, including commercially available software to partially meet the first two goals mentioned above. The proprietary product FLASH ID® (Sciometrics LLC, Chantilly, VA) is a software package that also relies on scanned images of written documents and produces graphical structures called *graphemes*. Graphemes are then grouped and sorted using an approach based on adjacency matrices (see Section 2.3) and linear discriminant analysis. To address the third goal, the algorithm searches a database for closest writing matches and gives results based on a scoring system. Two other alternatives are the WANDA workbench [5] and CEDAR‐FOX [14] systems, that have tools to facilitate both automated and interactive document examination, as well as database management frameworks.

Our work does not rely on any character recognition techniques. In that sense, it is reminiscent of other work presented in the literature. For example, in their 2007 paper Bulacu and Schomaker [3] use graphemes that are normalized to a 30 × 30 pixel image and overlaid to extract pixel shade differences. A standard clustering algorithm yields a code book, and grapheme distribution across that code book is one of six features used to identify writers in downstream writer verification analysis. Miller et al. [12], whose methods are “substantially similar to those used in the proprietary product FLASH ID,” group graphemes in a deterministic fashion, and measurement comparisons are made within those groups.

Our document analysis pipeline begins by processing scanned handwritten documents, and segmenting the writing into small graphical structures which we call graphs. Graphs are the smallest units of writing that we consider, and they often, but not always, correspond to letters and numbers. We group together graphs using measures based on similarity of major physical attributes. In this work, we solve the problem of grouping graphical structures through the development of a *K*‐means clustering algorithm that relies on a distance measure proposed by us and designed specifically for graphs. Just for comparison, we also develop our own deterministic grouping method, similar to that of Miller et al. [12]. The groupings that result from the dynamical clustering method we propose are more parsimonious, descriptive, and repeatable for writers than deterministic groupings, because of their robustness to small structural differences among graphs. This dynamic method of grouping better characterizes a writer's handwriting. When the vector of observed cluster frequencies contributed by a writer are the response variable in a Bayesian hierarchical model, the probability with which we can identify the writer of a document greatly improves over that resulting from deterministic groupings of graphs. Examples to illustrate the deterministic and the dynamic grouping approaches and to motivate the rest of the work follow below.

Consider two writers. Suppose that writer A favors formal cursive, so they tend to make loops when forming characters such as “l” and “f.” Conversely, writer B uses a more broken writing style and tends to make a single stroke “l” and loop‐less segments in an “f.” Ideally, when writer A's documents are processed, their graphs would be assigned to groups that are characterized by a higher rate of cursive style loops. When writer B's graphs are considered, their distribution over the groups should differ from that of writer A, since writer B will have fewer graphs that are characterized by loops, and more that are assigned to groups embodying simple stroke graphs.

**FIGURE 1:**
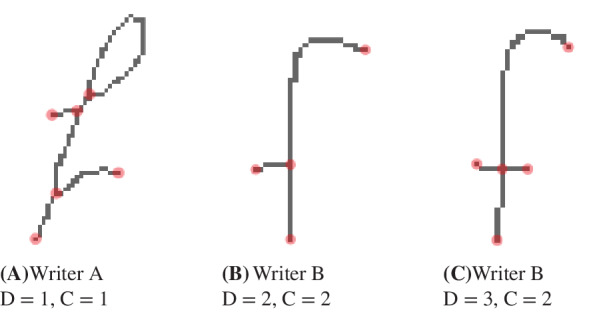
Three graphs whose assignment will contribute to characterizing a writer's style. D is deterministic group assignment. C is cluster group assignment

Figure 1 shows three example “f”s from two writers. The characters in Figure 1B, C are simple block “f”s (like writer B might produce), while Figure 1A shows a cursive style “f” with a loop near the top (like writer A might write). Deterministic and clustering based groupings of these three “f”s are provided in their captions as D and C, respectively. Notice that when compared to Figure 1C, Figure 1B is missing an appendage. This results in different deterministic group assignments for the two letters despite other clear structural similarities. This is unfortunate for an algorithm attempting to differentiate writer A from B based on the grouping.

Using the clustering method we propose, both Figures 1B, C are assigned to the same group (*C* = 2). Their overall structure is dominating and the small incidental edge that is missing from the rightmost “f” does not force a separation between groups. This results in a partitioning of the “f”s that can be used to help identify each character's writer. Of course, this is an example for illustration, with only one character type. In a real application there is a variety of graphical structures to group into clusters, and writer identification relies more on an aggregation of small gains across many groups, rather than on the hard division described here.

This paper is organized as follows. Section 2 discusses the document processing pipeline for taking a handwritten document from a scanned image to usable data (graphs). A deterministic grouping method is presented as a direct result of the graphical structures that arise from processing (Section 2.3). We introduce our clustering algorithm for graphs in Section 3. The resulting clustering template is used as a feature extraction device for a sample of writers from the CVL handwriting dataset [8] in Section 4. We compare writership analysis results for the dynamic cluster groupings to results obtained using the same model and writers, but based on deterministic groupings. Section 5 includes a summary of findings and suggestions for future work in this area.

**FIGURE 2:**
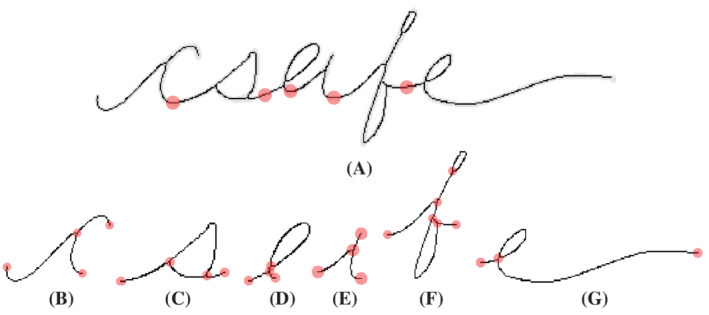
The word “csafe” taken from a processed document. (A) Binarized pen strokes in gray, pixel‐wide skeleton overlaid in black. Red dots denote graph breakpoints of the connected writing. (B–G) Each graph shown separately where red dots show nodes (endpoints and intersections)

## 2 SEGMENTING A DOCUMENT INTO GRAPHS

This section addresses the first of two feature extraction phases that occur in our analysis pipeline. This phase of document processing and extracting usable data begins with a scanned handwritten document and results in a set of *graphs*. Graphs are small pieces of connected ink that serve as individual observations for the analyses that follow. Processing is done using the R package handwriter, available at https://github.com/CSAFE‐ISU/handwriter. This R package, written by Nick Berry for the Center for Statistics and Applications in Forensic Evidence (CSAFE) at Iowa State University, provides a toolkit for handwritten document processing.

### 2.1 Preprocessing of a handwritten document

In the handwriter pipeline, preprocessing consists of several steps. First, the scanned document is *binarized* to convert an image to pure black and white. Colored images are turned to grayscale using the linear combination 0.2126*R* + 0.7152*G* + 0.0722*B* on each pixel. Otsu's binarization method [15] is applied to the grayscale images to assign each pixel to one of two groups in a fashion that maximizes between‐class variance. The groups are appropriately normalized to black and white.

The next step is to “clean” the image. Cleaning is done by handwriter using procedures from [20]. A set of masks are implemented that isolate and correct spurious pixels, fill white holes likely caused by mistakes in the binarization step, and widen holes that were likely intentionally made during writing. On the clean binary image we use the Zhang‐Suen thinning algorithm [24], to reduce the writing to a one pixel‐wide skeleton structure that maintains the shape and connections of the original. By choosing to work with the writing skeleton we sacrifice all information about the width of lines. However, thinning facilitates our ability to identify structural components in the handwritten images.

**FIGURE 3:**
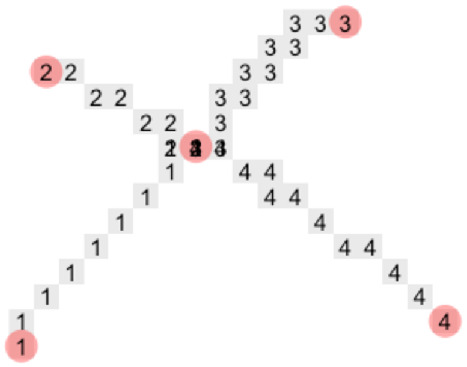
The letter “X” processed by handwriter. Numbers show different paths. Red dots (if in color) indicate nodes. Note that the middle node is actually a merging of two close by nodes

Figure 2A shows an example of a binary image displayed in gray with its thinned, pixel‐wide skeleton overlaid in black. The overall shape of the written text remains intact and key structural features (like terminal pixels, intersections, and the pixel‐wide paths connecting them) are easier to detect. Section 2.2 expands on the idea of using simple structural elements of the skeleton to extract requisite information from the documents.

**FIGURE 4:**
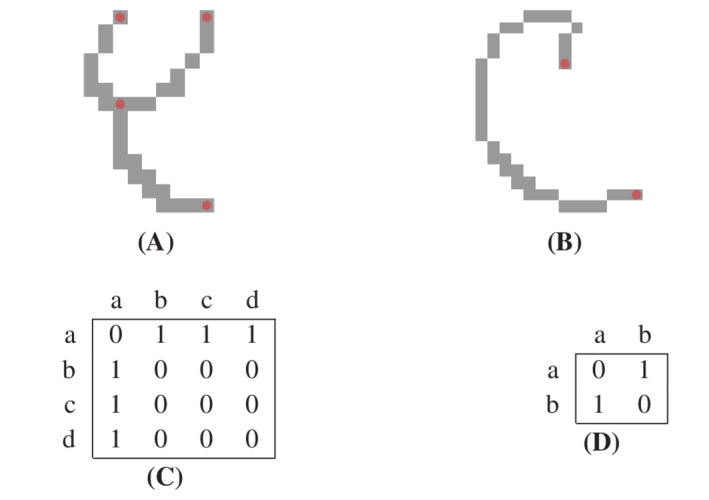
(A) An example of the most common graph structure. (B) An example of the next most common graph. (C and D) The corresponding adjacency matrices

### 2.2 Segmenting connected ink into graphs

After a document has been preprocessed as described above, the handwriter package decomposes the skeletonized writing into a sequence of disjoint graphs. Consider the *i*th graph of a document as an attributed graph $G_{i}$ with a set of vertices $V_{i}$ made up of the terminal pixels and locations of intersecting lines of the graph. The set of edges ℰ_*i*_ represents paths in graph *i* that connect elements of $V_{i}$. For the graph representing an “x,” shown in Figure 3, the red dots make up the set $V_{i}$, and each of the four paths (individualized by numbers) are elements of the set ℰ_*i*_. These graph structures, similar to those in [7, 12, 17, 23], will be the basis for evaluating the structural styles of a writer.

We start by breaking the writing down into a set of connected pieces of ink. In cursive writing this generally corresponds to a connected word, and in disjoint print, a single character. Individual connected ink blots are then candidates to be decomposed further into graphs.

By following a set of rules, handwriter makes the decision on which ink blots to break by looking through each edge in an ink blot and breaking those that satisfy the rules designed to estimate the intended character breaks within that ink blot. Breakpoints of the word “csafe” in Figure 2A are shown in red.

Once this step is completed, the document is fully decomposed into a disjoint set of graphs $\left\{G_{i}\right\}$, each with its respective vertices and edges. Finally, the vertices in each graph are systematically ordered so that we can compare vertices across graphs as discussed in the sections that follow. For example, with these ordered vertices and edges between them, we can construct an adjacency matrix for each graph as a means to characterize the graph, as described in Section 2.3.

It is worth mentioning that we only use handwriter for document processing in this work, but the software also has other feature extraction capabilities such as finding centroids, slants, loops, and other measurable attributes for each graph. These features are undeniably important for forensic handwriting analysis, but are not used to create the clustering template that is of focus here, and thus we do not discuss them further.

### 2.3 Adjacency grouping

We previously suggested that by establishing a grouping system for graphs, we can assess the rate at which a writer produces graphs of each group, and use those rates to characterize a writer's style. The first grouping method, which we will use for baseline comparison with our clustering method, uses only the edge connectivity of a graph. Graphs with identical adjacency matrices are placed together in a group. We call this the adjacency grouping method, and it is readily available after processing and segmentation by handwriter. This deterministic grouping method is sensitive to small changes in graph structure, because small incidental pen strokes change the adjacency matrix of a graph, and thus the adjacency group assignment. Since this method is so sensitive to small differences between the graphs, the number of resulting groups is very large. Walch and Gantz [23], Gantz et al. [7], Saunders et al. [17], and Miller et al. [12] utilize, in part, a similar method, which they call the “isocode,”

For illustration, we provide adjacency grouping outcomes based on 160 documents from the CVL database [8]. handwriter partitions these documents into a total of 52,889 graphs. The number of resulting unique adjacency groups is 1764. The two most common adjacency groups, shown in Figure 4 with graph examples, account for approximately 60% of all graphs in the CVL documents.

Advantages of this grouping method arise from the strict structural similarity imposed by the identical adjacency matrices between the members of a group. Within a group, each graph has the same number of edges and vertices, allowing for one‐to‐one comparisons between structural components of the graphs. Gantz et al. [7] leverage this advantage. On the other hand, the required strict similarity means that graphs with minor differences are not grouped together, are never compared, and this potentially leaves valuable information unused.

## 3 CLUSTERING ALGORITHM FOR GRAPHS

As discussed above, using the adjacency information to group similar graphs often results in a very large number of groups. When applied to a sample of documents such as those included in the CVL database, the vast majority of graphs end up in a small number of groups and the rest of the groups contain one or few observations. We develop an approach that will allow us to control the number of groups, each of which will hold graphs that may not be identical in connectivity, but have similar dominating structure.

We propose a dynamic and flexible *K*‐means‐based grouping method that is tolerant to incidental pen strokes. This allows graphs with similar, but not identical, graphical structures to be captured in the same group.

All *K*‐means‐type clustering algorithms hinge on the ability to calculate two essential quantities: a distance (or discrepancy, or similarity) measure and a measure of center. Neither of these measures are readily or easily defined for graphs. Section 3.1 summarizes the notion of an edit distance used in the general graph framework. Then, for graphs that represent handwriting, we develop a novel distance measure and a mean calculation. Finally, we outline an accompanying clustering algorithm.

### 3.1 Edit distances

An existing discrepancy measure for graphical structures, which helps to motivate ours, is the error‐correcting graph matching of [11]. Their distance measure for two structures is the cost associated with a sequence of steps necessary to transition from one graph to the other, called the *edit distance*. This graph edit distance is related to the simpler concept used to quantify the difference in two strings [9]. To obtain the string edit distance, a set of operations is sequentially applied to a string *S*_1_ until it matches another string *S*_2_. The available edit operations are *Change*, *Insert*, and *Delete*. The resulting distance calculation can be posed and solved as a dynamic programming problem [22].

In the graph edit distance calculation, as in the case of strings, the goal is to sequentially apply edit operations to transition one graph to another. The necessary operations for graph editing are conceptually the same as in the string context, except that they can be applied to both edges and vertices. Each edit operation is assigned a cost, and the difference between two graphs is the sum of the minimal cost edit sequence between them.

Graphs that are compared using this method have labeled vertices, but generally do not have edge attributes such as lengths or curvatures, or vertex attributes like locations. The graphs that describe handwritten structures do have these attributes, which will be leveraged in their distance calculations. In the following section, we exploit edge attributes to develop a distance measure for two graphs and a mean calculation for a set of graphs. These measures will emulate aspects of the graph edit distance measure, with costs corresponding to the magnitude of the changes necessary to transition one graph into the other.

### 3.2 Distance measure for graphs

There are distinct differences in how distances are approached for handwritten graphs, rather than the general edit distance mentioned above. First, only edges of the graphs are taken into account. There is no cost directly associated with vertex edits. Every edge has two vertices, so differences in the vertices of the graphs can be reflected by alterations of the edges. Second, the attributes of the edges will be used to determine the cost associated with each change.

Each graph is completely described by its collection of edges, so the task of calculating graph distances can be simplified to calculating edge distances. In the section to follow, we present a distance measure for two edges, then provide the mechanism for computing full graph distances by combining edge distances.

#### 3.2.1 Distance between two edges

To develop the edge distance measure we keep in mind that the distance between graphs should be, at least in part, characterized by their structures, while allowing graphs with similar but not identical structures to be grouped together.

For two edges, the edge distance calculation is comprised of three component distances, *d*_*loc*_, *d*_*sld*_, and *d*_*sh*_, capturing the difference in endpoint locations, difference in the straight‐line distance between edge endpoints, and a rough estimation of the difference in edge shapes respectively. Each of these components will be addressed in turn.

**FIGURE 5:**
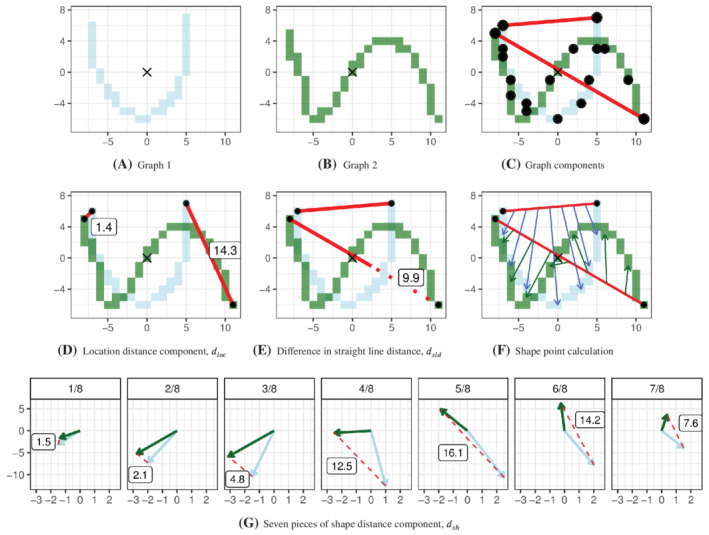
Edge distance measure calculation companion figures

To allow for meaningful comparisons of the locations of edges, we first align graphs on the 2‐dimensional coordinate system with their centers of gravity overlapping on the origin. Figure 5A, B shows single edge graphs on an *x*, *y* grid, each with their centroids anchored at the origin.

Note that the starting and ending points of edges are labeled arbitrarily, so the edge comparison will need to be considered in both traversal directions. When end point ordering is relevant for the three component calculations, a plus sign subscript, *d*_+_, indicates a distance component taken in the original assignment order of the paths, and a minus sign subscript, *d*_−_, indicates that the order of the second path is considered in reverse. Choosing the correct order of comparison will be addressed in Equation (3) after all three of the edge distance components have been established. Only the positive traversal comparisons are pictured in Figure 5, as that turns out to the be the proper comparison direction for these two edges.

We begin by defining the endpoint location component of the edge distance, *d*_*loc*_, which only requires the pixel positions of the end points of the paths. Denote the position of the first terminal pixel, the starting point of an edge, *e*, as $p_{1}^{e}$. The second terminal pixel position, the end point of the edge, is $p_{2}^{e}$. These locations are recorded as (*x*, *y*) points on the coordinate plane. The points connected by red lines in Figure 5C represent the edge end points.

Let ‖⋅‖_2_ denote the Euclidean norm. Then, the location component is defined as, *d*_*loc*_(*e*_1_, *e*_2_) = {*d*_*loc*+_(*e*_1_, *e*_2_), *d*_*loc*−_(*e*_1_, *e*_2_)}, where

$$\begin{array}{ll} & d_{\mathrm{loc}+}\left(e_{1},e_{2}\right)=\min\left\{{\left\|p_{1}^{e_{1}}-p_{1}^{e_{2}}\right\|}_{2},{\left\|p_{2}^{e_{1}}-p_{2}^{e_{2}}\right\|}_{2}\right\}\\ & d_{\mathrm{loc}-}\left(e_{1},e_{2}\right)=\min\left\{{\left\|p_{1}^{e_{1}}-p_{2}^{e_{2}}\right\|}_{2},{\left\|p_{2}^{e_{1}}-p_{1}^{e_{2}}\right\|}_{2}\right\}. \end{array}$$

In this formula, any two paths that either begin or end at the same pixel location will have a *d*_*loc*_ = 0 regardless of their shapes, angles, or lengths. Figure 5D shows ${\left\|p_{1}^{e_{1}}-p_{1}^{e_{2}}\right\|}_{2}=1.4$ and ${\left\|p_{2}^{e_{1}}-p_{2}^{e_{2}}\right\|}_{2}=14.3$, resulting in *d*_*loc*+_(*e*_1_, *e*_2_) = 1.4.

The second and third components will make use of the straight line that connects $p_{1}^{e}$ and $p_{2}^{e}$ in an edge, *e*, which we denote as ℓ^*e*^. The red line segments in Figure 5C depict $\ell^{e_{1}}$ and $\ell^{e_{2}}$ for the two example single edge graphs.

The second distance component represents the difference in the lengths of $\ell^{e_{1}}$ and $\ell^{e_{2}}$. This component adds a penalty reflecting the difference in the straight line displacement of the endpoints in each edge. Define this straight line distance component *d*_*sld*_(*e*_1_, *e*_2_) as

$$d_{\mathrm{sld}}\left(e_{1},e_{2}\right)=\left|{\left\|\ell^{e_{1}}\right\|}_{2}-{\left\|\ell^{e_{2}}\right\|}_{2}\right|.$$

In this case there is no need to make this comparison in both directions since the specification of the straight line between end points for an edge, ℓ^*e*^ is unchanged by the ordering of the terminal pixel locations $d_{1}^{e}$ and $d_{2}^{e}$. The diagonal segment in Figure 5E depicts this component for our example edges. The solid portion has the same length as the shorter segment, and the dotted portion, with a length of 9.9, represents the difference in the distances of $\ell^{e_{1}}$ and $\ell^{e_{2}}$.

For the third component of edge distance, we define a set of points that capture the shape of an edge. Denote the seven points on the edge *e* that cut it into eight equal length pieces as $q_{1}^{e},q_{2}^{e},q_{3}^{e},\ldots ,q_{7}^{e}$. Similarly, place seven equally spaced points on the straight line that connects the edge endpoints and call them $q_{1}^{\ell },q_{2}^{\ell },q_{3}^{\ell },\ldots ,q_{7}^{\ell }$. Then, subtract each of the line points from each of the corresponding edge points to obtain seven calculated shape points that are a rough representation of the shape of edge *e*. These shape points are $s_{1}^{e}=q_{1}^{e}-q_{1}^{\ell },\ldots ,s_{7}^{e}=q_{7}^{e}-q_{7}^{\ell }$.

The shape points capture the direction and magnitude of the edge's deviation from the straight line ℓ^*e*^. If an edge is relatively straight (think of the number one), we do not expect to see much deviation from ℓ^*e*^. If an edge has more curvature (think of the number five) we expect more deviation of the edge from the straight line.

We take *d*_*sh*_ as the average difference in the corresponding shape points of *e*_1_ and *e*_2_. Here, we certainly need to consider both the forward (*d*_*sh*+_) and backward (*d*_*sh*−_) orderings of the shape points. The shape contribution to the edge distance measure *d*_*sh*_(*e*_1_, *e*_2_) = {*d*_*sh*+_(*e*_1_, *e*_2_), *d*_*sh*−_(*e*_1_, *e*_2_)} is

(1) $$d_{\mathrm{sh}+}\left(e_{1},e_{2}\right)=\frac{1}{7}\left[{\left\|s_{1}^{e_{1}}-s_{1}^{e_{2}}\right\|}_{2}+\cdots +{\left\|s_{7}^{e_{1}}-s_{7}^{e_{2}}\right\|}_{2}\right]$$

or

(2) $$d_{\mathrm{sh}-}\left(e_{1},e_{2}\right)=\frac{1}{7}\left[{\left\|s_{1}^{e_{1}}-s_{7}^{e_{2}}\right\|}_{2}+\cdots +{\left\|s_{7}^{e_{1}}-s_{1}^{e_{2}}\right\|}_{2}\right].$$

In Figure 5F, vectors are drawn from the line points to the edge points, depicting the formulation of the shape points *s*_1_ through *s*_7_ for each edge. Figure 5G shows the same vectors, pointing at each of the seven shape points for each edge. The distance between shape points in each panel of Figure 5G is taken (shown next to the red lines), and the average of those seven distances make up the shape component of the edge distance. Here,

$$d_{\mathrm{sh}+}=\frac{1}{7}\left(1.5+2.1+4.8+12.5+16.1+14.2+7.6\right)=8.4.$$

Now, with all three edge comparison components (*d*_*loc*_, *d*_*sld*_, and *d*_*sh*_) in‐hand, the final step is to combine them into a single distance metric. We must take care to scale the edge distances so edges that make up a large proportion of their graphs are weighted more heavily. This gives way to an edge distance calculation that is robust to differences in small edges, but demands that large edges be similar. This is a desirable property since larger edges generally dominate the structure of a graph, and to find similar graphs, we seek similar dominating structures. By down‐weighting the small edge distances we introduce tolerance and prevent small edges from drastically influencing the distance measure. The amount that an edge distance is down‐weighted is based on the average proportion of the two graphs that the edges *e*_1_ and *e*_2_ make up.

The distance measure between two edges *d*(*e*_1_, *e*_2_) is obtained by combining the three components and weighting results. It is calculated as

(3) $$\begin{array}{cc} & d\left(e_{1},e_{2}\right)=\frac{1}{2}\left[\frac{\left|e_{1}\right|}{\sum_{i=1}^{|{ℰ}_{1}|}\left|e_{i}^{{ℰ}_{1}}\right|}+\frac{\left|e_{2}\right|}{\sum_{i=1}^{|{ℰ}_{2}|}|e_{i}^{{ℰ}_{2}}|}\right]\\ & \;\times \min\left\{d_{\mathrm{loc}+}+0.5d_{\mathrm{sld}}+2d_{\mathrm{sh}+},d_{\mathrm{loc}-}+0.5d_{\mathrm{sld}}+2d_{\mathrm{sh}-}\right\}, \end{array}$$

where we let $e_{i}^{{ℰ}_{j}}$ denote the *i*th edge in ℰ_*j*_, the set of edges comprising graph *j*. The leading fraction in (3) is the weighting component, where |*e*_*i*_| denotes the length of path *i* and |ℰ_*j*_| is the total number of edges in graph *j*. The minimum operator in the last component of Equation (3) is the mechanism for choosing the direction of edge comparison that yields the smallest distance measure.

The edge distance calculation relates to fundamentals of the graph edit distance. The endpoint location component *d*_*loc*_ emulates an edit operation that shifts an edge in space. The cost of this edit is equivalent to the distance that an edge must travel to align itself with its counterpart's nearest endpoint. The straight‐line distance component *d*_*sld*_ measures the difference in displacement of the edge endpoints. This is comparable to stretching or compressing edges as an edit operation. The final component *d*_*sh*_ represents the amount that two paths have to be straightened, curved, or twisted in order to match each other.

#### 3.2.2 Graph distance measure from edge calculations

In Figure 5 the edge distance is equal to the graph distance since each graph has only one edge. For more complicated graphs, combining edge distances to form a complete graph distance happens in two phases. First, we need to address potential differences in edge counts between two graphs. Then, the optimal matching between the edge sets is chosen via evaluation of all available edge distance comparisons. The edge distances for the resulting matches are summed to form the final graph distance measure.

To equalize the number of edges between two graphs, dummy edges are added to the graph with smaller |ℰ| until the number of edges is equal. These dummy paths do not have any physical structure, and thus cannot be compared with real edges in the usual way (using endpoints, shape points, etc.). Instead, the distance between a real path and a dummy path is assigned solely based on the length of the real path. For a real edge *e*_*r*_ and a dummy edge *e*_*d*_, we define their distance as

(4) $$\begin{array}{ll} d\left(e_{r},e_{d}\right) & =\frac{1}{2}\left[\frac{\left|e_{r}\right|}{\sum_{i=1}^{\left|{ℰ}_{r}\right|}\left|e_{i}^{{ℰ}_{r}}\right|}+\frac{0}{\sum_{i=1}^{\left|{ℰ}_{2}\right|}\left|e_{i}^{{ℰ}_{2}}\right|}\right]{\left|e_{r}\right|}^{2}\\ & =\frac{1}{2}\left[\frac{{\left|e_{r}\right|}^{3}}{\sum_{i=1}^{\left|{ℰ}_{r}\right|}\left|e_{i}\right|}\right]. \end{array}$$

This calculation uses the same weighting factor as in Equation (3) with |*e*_2_| = 0 and the distance component set to the squared length of the real path, *e*_*r*_.

By including the dummy edge comparisons, we make max(| ℰ_1_| , | ℰ_2_| )^2^ unique pairwise edge distance calculations for $G_{1}$ and $G_{2}$. We match the edges in $G_{1}$ with edges in $G_{2}$ in a one‐to‐one fashion such that the total edge distance is as small as possible. This task can be formulated as a constrained minimization optimization problem, which is solved via linear programming.

We now give a formal expression for the complete graph distance measure. Define $I^{*}=\left\{i_{1}^{*}\ldots i_{\max\left\{\left|{ℰ}_{1}\right|\left|{ℰ}_{2}\right|\right\}}^{*}\right\}$ to be the set of indices that reorder the edges of $G_{2}$ to reflect the optimal minimum distance matching with respect to the original ordering of $G_{1}$. The distance between the two graphs is

(5) $$D\left(G_{1},G_{2}\right)=\sum_{i=1}^{\max\left\{\left|{ℰ}_{1}\right|,\left|{ℰ}_{2}\right|\right\}}d\left(e_{i}^{{ℰ}_{1}},e_{I_{i}^{*}}^{{ℰ}_{2}}\right).$$

### 3.3 Weighted mean of graphs

We begin by defining a weighted mean of two graphs. Once this procedure has been established, it can be applied in sequence to produce the mean of a larger set of graphs by iteratively computing weighted means with decreasing weight on each newly introduced graph. Just as the graph distance measure relied on combining individual edge distances, the graph mean calculation will rely on combining individually calculated edge means. This procedure requires that the edges of two graphs have been matched such that they provide a minimum graph distance calculation as described in Section 3.2.2.

With the exception of dummy edges, an edge is characterized by just its end points, shape points, and length, so the weighted mean of two edges is constructed as the weighted mean of each of these components. The heavier the weight on the first graph, the closer all of the mean edge components will be to that graph's end points, shape points, and length.

**FIGURE 6:**
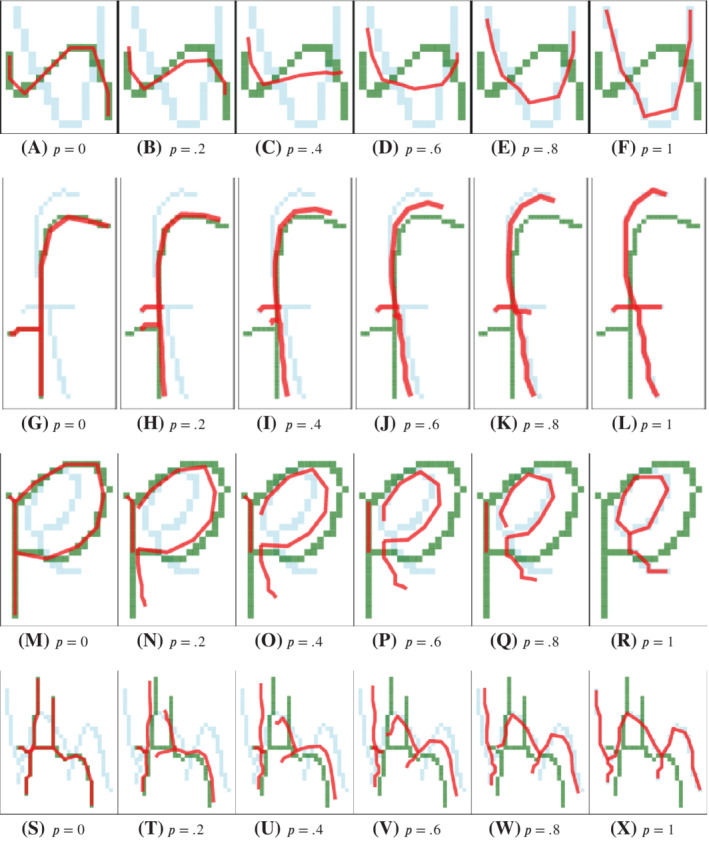
Figures showing weighted means of two letters. The red line represents the weighted mean, and the weighting component, *p*, indicates the amount of weight placed on the blue graph in each panel. For example, (A) shows the mean weighted completely on the “n,” then left to right transitions from “n” to “v,” and finally (F) shows the mean weighted completely on the “v”

Figure 6A–F demonstrates the steps in the calculation of a weighted mean in the simple situation in which both graphs have one edge. Notice that as the weight on the blue graph grows, the endpoints, seven shape points, and length of the red mean are pulled towards the blue graph.

When one of the edges in the mean calculation is a dummy edge, the mean must be calculated in a slightly different way. A real edge that is matched with a dummy edge lends its spatial components to the mean edge, so the resulting mean has the same end points and shape points as the real edge. However, the length of the resulting edge is a weighted average of the length of the real edge and 0, down weighting that edge's importance in the means.

Figure 6G–L shows the weighted mean of two graphs that have a different number of edges. In this example, the crossing on the green “f” is matched with the crossing edge on the right side of the blue “f” as result of the linear programming routine. The dummy edge appears in Figure 6H when the blue graph begins to accumulate some weight, where it is matched with the remaining real edge. Although the images do not show it, the length of that edge is down‐weighted between the length of the blue edge it represents and 0 (the length of the dummy edge).

Due to repeated down‐weighting of an edge across a set of graphs, it is possible that the length of certain edges will get very small. We delete any edge from the mean graph that is shorter than 1 pixel long in the sense of weighted length. This prevents the number of edges in the mean graph from being equal to the largest graph in the set. Figure 6M–X illustrates a more complex comparison where dummy edges and pruning both play a role as the weightings progress towards *p* = 1.

With this process we can calculate the mean of two graphs for any value of the weight, *p*. Now consider a larger set of graphs, say $G=\left\{G_{1}\ldots G_{n}\right\}$. Define a function to calculate the weighted mean $w^{\prime }\left(G_{1}G_{2}p\right)$ where *p* is the [0, 1] weight placed on $G_{1}$. Then, the mean of the first two elements of G is $m_{2}=w^{\prime }\left(G_{1}G_{2},0.5\right)$. To incorporate the third element into the mean, we must take care that the included graphs are each weighted evenly in *m*_3_, so $m_{3}=w^{\prime }\left(m_{2},G_{3},\frac{2}{3}\right)$. For each subsequent mean calculation the update formula is $m_{i}=w^{\prime }\left(m_{i-1},G_{i},\frac{i-1}{i}\right)$. Applying this update formula iteratively across a set of graphs provides a method for calculating the mean of a set of graphs.

While this set mean does a good job of summarizing a set of similar graphs, it has some properties that are suboptimal when encountering a set of widely varying graphs. The most egregious is that the mean of the set of graphs depends on the order in which the graphs are introduced into the update formula. This is due to the path matching step of the algorithm, and is unavoidable. We address this shortcoming in the next section where we modify the measure of center that is used in the clustering algorithm.

### 3.4 *K*‐means‐type algorithm

Before we implement the *K*‐means framework, we address the question of outliers in the context of our data format. The presence of outliers can have undesirable effects on cluster groupings [21] in any setting. In out setting, outliers might arise when, for example, the document includes crossed‐out words. These outliers will be represented by graphs that are very different from all of the others. Additionally, there may be sets of similar graphs that do not occur frequently enough to form their own cluster, but are not close to any of the other cluster centers. Such observations are also considered outliers in our setting.

**FIGURE 7:**
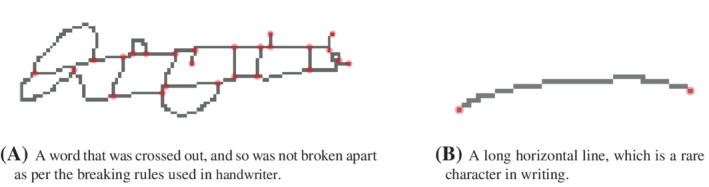
Two very different graphs that will be considered part of an outlier group during the clustering routine

Figure 7 shows two examples of unconventional graph types that will be far from every cluster mean. Figure 7A is a large complex graph that is not repeatable, and Figure 7B is a simple, but uncommon structure in handwriting. When *K*‐means encounters an observation very far from any cluster center it may either pull the center of a cluster towards it, thereby summarizing the rest of the cluster poorly, or it may create its own cluster with only that point. Neither of these options are desirable.

To accommodate outliers, we implement the method of Gan and Ng [6]. A cutoff distance is derived from the average distance from each observation to its cluster mean, and used to decide which are too far from any center. Such observations are grouped as a set of outliers, meaning they will not contribute to any cluster center calculations. The number of outliers is capped by a parameter, but the number of classified outliers is learned based on the data. In the case of no outliers the modified algorithm simplifies to standard *K*‐means.

The clustering framework of Forgy [4] and Lloyd [10], the simplest formulation of *K*‐means, starts with a set of initial clusters and iterates through two steps until convergence. First, each point in a dataset is assigned to the cluster whose center is closest to it, then the cluster centers are recalculated with all of the points that were just assigned to that cluster. Rather than use the cluster means calculated as in Section 3.3, we increase the stability of the clustering algorithm by using the cluster *exemplar* as the center for the group reassignment step. A cluster exemplar is simply the actual data point closest to the calculated cluster mean. The complete algorithm is as follows.

**Outlier tolerant *K*‐means algorithm for graphs**

Consider a set of observed graphs ***X*** = {*X*_1_, …, *X*_*n*_}, a pre‐specified number of clusters ***K***, a set of initial cluster exemplars $C=\left\{C_{1}\ldots C_{K}\right\}$, a maximum number of outliers *n*_*o*_, and a parameter *γ* which is part of the calculation that controls the allowable distance from a center to an observation before it is classified as an outlier. Also define the variable *T*_*o*_ to be the distance threshold by which outliers are determined. *T*_*o*_ will be learned and adjusted within each iteration of the algorithm, but the initial value of *T*_*o*_ = ∞. The cluster assignment of observation *i* is *φ*_*i*_, where *φ*_*i*_ =  − 1 denotes that the *i*th observation is an outlier.

Iterate through the following steps until the cluster assignments do not change:

1. Assign each graph *X*_*i*_ ∈ **X** to the cluster whose exemplar is nearest to *X*_*i*_ with respect to the distance measure in Section 3.2. Let the assignment be defined as $\varphi_{i}=\arg\min_{j=1,\ldots ,K}\left\{D\left(X_{i}C_{j}\right)\right\}$.
2. Call the graph an outlier (set *φ*_*i*_ =  − 1) if its distance to its cluster exemplar, $D\left(X_{i}C_{\varphi_{i}}\right)$, is larger than *T*_*o*_. If more than *n*_*o*_ observations would be outliers, only reassign the observations with the *n*_*o*_ largest distances.
3. Calculate the mean of the graphs in each cluster as in Section 3.3. To facilitate stable convergence of the algorithm, we calculate the iterative average by including graphs in order of their ranked distances to the exemplar. Outlier graphs do not contribute the mean calculations. Update $C_{1},\ldots ,C_{K}$ to be the *X*_*i*_ closest to each calculated mean (exemplars) via $C_{j}=\arg\min_{X_{i}|\varphi_{i}=j}D(X_{i},\text{Mean}\left(\left\{\left.X_{m}\right|\varphi_{m}=j\right\}\right)$.
4. Update *T*_*o*_ with

$$T_{o}=\frac{\gamma }{n-\sum_{i=1}^{n}I\left(\varphi_{i}=-1\right)}\sum_{i=1}^{n}I\left(\varphi_{i}\neq -1\right)D\left(X_{i},C_{\varphi_{i}}\right).$$

When the algorithm has converged, each graph is assigned to the cluster with the nearest center, with the exception of the outlier cluster. The final set of exemplars is stored as a template so that graphs extracted from future documents can be classified according to the exemplar they are most similar to. Like all versions of *K*‐means, only a local optimum is guaranteed. Running the algorithm with different starting values is suggested to try to reach the global optimum. The input parameters *n*_*o*_ and *γ* are set with the default values of *n*_*o*_ = .25*n* and *γ* = 3, which allows for up to 25% of the observations to be called outliers and requires a distance three times the average within cluster distance to call an observation an outlier.

For this algorithm, *K* is assumed known and fixed before running. The purpose of clustering in this application is to find reasonable templates to be used as feature extraction tools, not to find the number of clusters that best partitions a set of graphs. The number of clusters we use is determined by the accuracy of the writer identification task, using the clustering template as a feature extraction tool for those data. It is not beneficial to find and employ a more parsimonious template if the predictions of the writer identification model are relatively unchanged by using different template sizes. We have explored reasonable choices for *K* to provide evidence that the choice does not have a large impact on the performance of the writership analysis. Details are presented in the following section.

## 4 APPLICATION

We demonstrate the use of our clustering method in a forensic handwriting analysis context using a subset of the publicly available CVL handwriting database [8] to perform a writership analysis on a set of documents with known origins. We use two partitions of the CVL database to demonstrate our methods. The first is used to create the clustering template that will later serve as a feature extraction tool. It is comprised of 54 writing samples from 27 writers. Each of the 27 writers contributed 6 writing prompts, 5 in English and 1 in German. We randomly select 2 English prompts from each writer to create the 54 sample dataset for the clustering algorithm. The second partition is comprised of 160 writing samples from 40 writers, distinct from the writers included in the first partition. Each of these writers contributed four writing samples, and we include three in the training dataset to estimate the parameters of the Bayesian hierarchical model that outputs writership probabilities. We set aside the fourth writing sample of each writer in the second partition to create a holdout set of documents. These will serve as questioned documents in the writer identification exercise.

Prior to use, documents from both partitions are cropped to the smallest bounding box containing writing and down sampled to 72 pixels, or dots, per inch (dpi) without changing the aspect ratio of the document.

### 4.1 Creating a clustering template

Graphs are extracted from the 54 documents belonging to the first partition of CVL data, as described above, and used as observations for the clustering algorithm. The algorithm was executed 20 times using each of *K* = 30, 40, 50, and 60, for a total of 80 executions. We consider more clusters than letters in the alphabet because there are a variety of forms for many letters that often correspond to graphs. For each choice of *K*, one template is selected by choosing the set of exemplars that provide the smallest within cluster sum of squares for the data. We will focus now on the template selected for *K* = 40.

**FIGURE 8:**
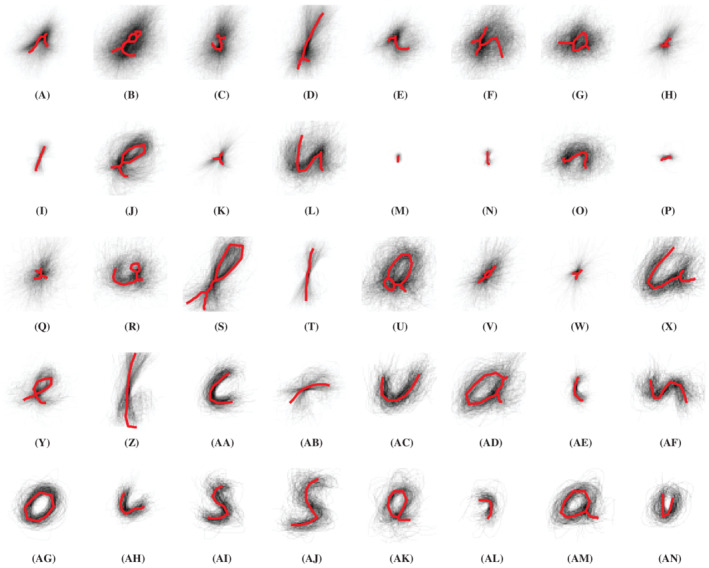
Each subplot represents 1 of the 40 clusters obtained from 54 CVL documents. The black background figures are the graphs which were grouped into the cluster at the final iteration of the algorithm. Exemplars are shown in red on top of the graphs

**FIGURE 9:**
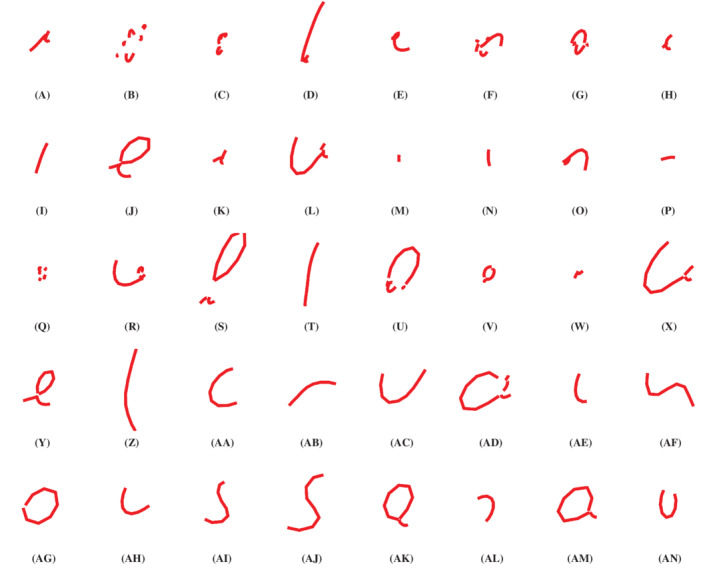
These plots show the calculated mean for each of the clusters at the final iteration of the algorithm. The panes are ordered to match the exemplars in Figure 8. These means indicate generally important characteristics of each cluster

Figure 8 shows 40 exemplars that comprise the *K* = 40 template, ordered based on the number of observed graphs that fall into each cluster, and Figure 9 shows the calculated means for the clusters in the same order. While the exemplars are used for functional purposes, the combination of these two measures of center provides an interesting point of view to investigate behaviors of the clusters built from handwritten documents. For brevity, we will not discuss each of the 40 clusters individually. Instead, we note some general trends and focus on specific clusters of interest.

Clusters like (i), (m), (n), (p), (t), and (ab) provide groups for simple single edge graphs of varying lengths and orientations. These clusters are generally highly populated, and contain graphs that are likely not very informative about the writer who created them. Clusters that are comprised of more complicated graphs have more natural variability both within and between writers. Clusters like (j), (s), (u), and (y) present with vertical loops ascending above the graphs, incorporating letters like cursive “l”s, ”e”s, and “d”s. Clusters like (a), (e), (h), (q), and (w) show small graphs with crossings, possibly representing letters like “k,” “r,” or ”x,” or cursive letters like “i” and “r.” These clusters also likely include graphs that come from only a piece of a letter that was broken apart by the rules defined in Section 2.2.

Some clusters clearly contain particular letters. Clusters (ai) and (aj) show forms of the printed letter “s.” Clusters (ad), (ak), and (am) show different ways to make the letter “a.” Clusters (f), (l), (o), (r), (u), (ac), (af), (ah), and (an) likely contain mostly “u”s, “n”s, and “h”s for print writers of various shapes and sizes.

One characteristic of Roman characters that is seemingly missing a dominant presence in any of the cluster exemplars is the descending stroke like that found in “g”s, “j”s, “q”s, or “y”s. The characters with descending segments tend to appear in clusters like (b), (c), and (u). It is not clear from the exemplars why these characters would belong in those clusters, but in Figure 9 there are small u‐shaped segments in the mean graphs of these clusters that match with the descending segments.

**FIGURE 10:**
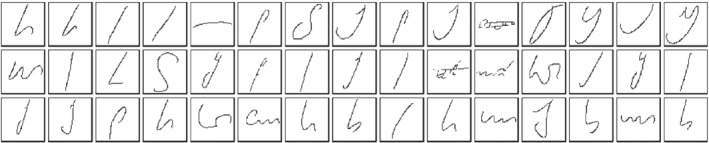
This set of graphs represent the observations categorized as outliers. Some of the outliers are very complex graphs, while more are large, simple graphs. The general trend for outliers is that they are large, simple graphs

We expected that large, non‐repeatable, complex graphs would be called outliers. This is true to some extent, but many of the complicated graphs are assigned to clusters with many edges instead. The cluster members and exemplar shown in Figure 8B show an example of this unexpected, but not unwelcome cluster type. From Figure 9B we observe that the mean contains some points scattered around the character space, which match well with and absorb the graphs with a large number of edges, making it very diverse. In addition to complex structures, a large proportion of outliers are actually simple graphs. See Figure 10 for examples of graphs considered outliers. The simple graphs classified as outliers tend to be very large in comparison to the other characters, and do not line up well enough with any of the existing cluster exemplars.

**FIGURE 11:**
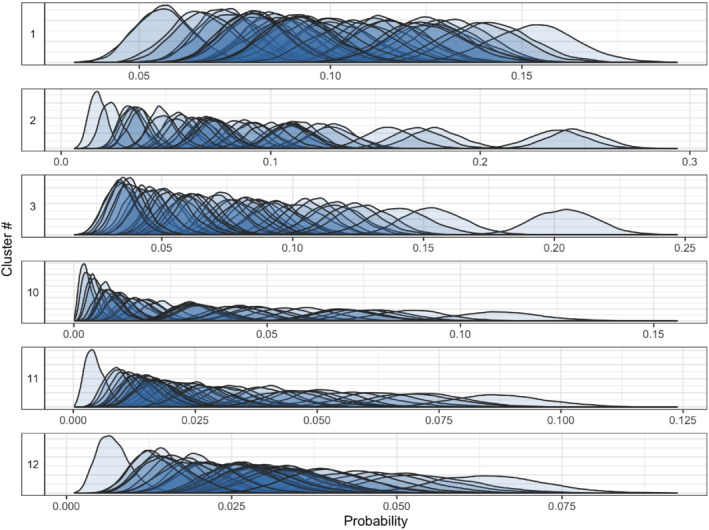
Posterior density estimates for *π*_*w*, *c*_, elements of the multinomial probability vector. Shown for known training writers *w* = 1, …, 40 and clusters *c* = 1,2,3,10,11,   and 12 (the 1st–3rd and 10th–12th most frequently occurring clusters). Panes are labeled with the cluster number they correspond to, and include one density for each writer

### 4.2 Writer identification

For the task of writer identification, we use three training documents from a closed set of 40 known writers. We first extract the graphs from the scanned documents using handwriter and each graph is then assigned to a clustor via comparison to the clustering template. To do this, the distance between a graph and each exemplar in the template, shown in red in Figure 8, is calculated using the distance measure defined in Section 3.2. The cluster assignment feature for the graph is the cluster number corresponding to the exemplar that produces the smallest distance comparison. For each document in the training dataset, we tally the number of graphs that are assigned to each of the *K* cluster exemplars. The notion of gathering handwriting information in “bins” has been used before (Bhardwaj et al. [2] use such data with a latent Dirichlet allocation model and Saunders et al. [17] with a variety of classifiers). The *K*‐dimensional vector of cluster frequencies we obtain for each document in the training set is used as the multinomial response variable in the hierarchical model we define in the next section. For each writer in the closed set, we then obtain an estimate of the posterior distribution of the vector of multinomial parameters.

We then turn to the set of holdout or “questioned” documents that we pretend have an unknown source, and try to find the most likely writer among those in the closed set. The posterior predictive probability of writership of a questioned document estimated for each writer relies on the multinomial parameter values learned about each known writer. The desired outcome is a probabilistic conclusion about writer identification for each of the held out documents. Ideally, the true writer of each of the questioned documents will have the highest posterior probability of writership. We compare results obtained from computing these writership probabilities using our dynamic cluster groupings with those obtained using the same model, but with the adjacency grouping method discussed in Section 2.3.

We will index model elements that correspond to writers from 1 to 40 for simplicity, but note that the writer identifiers from the CVL database are not consecutive, and we are using data from writers with identifiers that range from 52 to 176.

#### 4.2.1 Model formulation

Let *Y*_*w*(*d*), *c*_ be the number of graphs assigned to cluster *c* for training document *d* nested in writer *w* where,

- *c* = {1, …, *K*}, where *K* denotes the fixed number of clusters in the template, and the cluster ordering is the same as in Figures 8 and 9,
- *w* = 1, …, 40, and
- *d* = 1,2,3.

Then, ***Y***_*w*(*d*)_ = {*Y*_*w*(*d*), 1_, …, *Y*_*w*(*d*), *K*_} characterizes the number of graphs assigned to each cluster for document within writer, *w*(*d*). We consider these cluster assignments as samples from a multinomial distribution with a writer specific parameter vector, ***π***_*w*_, that captures rate at which a writer emits graphs that are most similar to each cluster exemplar. Let ***π***_*w*_ = {*π*_*w*, 1_, …, *π*_*w*, *K*_} denote the *K*‐dimensional simplex for writer *w* from a Dirichlet distribution. The *K* hyperparameters of the Dirichlet distribution, denoted as ***α*** = {*α*_1_, …, *α*_*K*_}, are assigned independent gamma prior distributions. The mathematical formulation of the model is

(6) $$\begin{array}{ll} & Y_{w\left(d\right)}\overset{\mathrm{ind}}{∼}\text{Multi}\left(\pi_{w}\right),\\ & \pi_{w}\overset{\mathrm{ind}}{∼}\text{Dirichlet}\left(\alpha \right),\\ & \alpha_{c}\overset{\mathrm{iid}}{∼}\text{Gamma}\left(a=2,\;b=0.25\right). \end{array}$$

Markov chain Monte Carlo (MCMC) estimates were obtained using the *rstan* R package [19]. In the analysis that follows, 4000 draws of each model parameter were collected after a burn‐in period of 1000. Denote these draws as ***α***^(*m*)^ and $\pi_{w}^{\left(m\right)}$, for *m* = 1, …, *M* (=4000).

Figure 11 shows posterior densities of the *π*_*w*, *c*_ components for all writers and six of the *K* clusters. Keep in mind that for every writer, the density in a pane represents one element of a 40‐dimensional simplex, and that during modeling, all of the ***π***_*w*_ elements are considered simultaneously. In this application, a perfectly discriminating feature would be one where there is no density overlap. Of course, there is no one cluster that satisfies this definition. However, a writer's style can be sufficiently captured and characterized by the rate in which their graphs are assigned to various clusters, jointly.

#### 4.2.2 Prediction

We use the model defined in Equation (6) to evaluate the writership of a questioned document with respect to the known writers in the closed set for which model parameters were estimated. Let the questioned document be written by unknown writer w^*^. Then, the multinomial response vector for the new document is $Y_{w^{*}}^{*}=\left\{Y_{w^{*},1}\ldots Y_{w^{*},K}\right\}$. We drop the “document nested within writer” notation, *w*(*d*), because we consider only one questioned document at a time.

The posterior predictive distributions are used to calculate probability of writership of the questioned document for each of the 40 writers in the training data. Consider a particular writer, *w*^′^, from the closed‐set of training writers. The goal is to estimate the probability that this particular writer was the author of the test document. Let $\pi_{w^{\prime }}^{\left(m\right)}$ denote the *m*th MCMC sample of the *w*^′^ multinomial parameter vector, where *m* = 1, …, *M*. Evaluate the multinomial likelihood under writer *w*^′^ at MCMC iteration *m* as

(7) $$q_{w^{\prime }}^{\left(m\right)}=\text{Mult}\left(Y_{w^{*}}^{*};\pi_{w^{\prime }}^{\left(m\right)}\right).$$

The evaluation is conducted for all 40 known writers in the closed‐set and stated together as

(8) $$q^{\left(m\right)}=\left[q_{1}^{\left(m\right)},q_{2}^{\left(m\right)},q_{3}^{\left(m\right)},\ldots ,q_{40}^{\left(m\right)}\right],$$

and at MCMC iteration *m*, we recognize the writer with the largest likelihood evaluation with a “vote”,

(9) $$v_{w^{\prime }}^{\left(m\right)}=\left\{\begin{array}{ll} 1 & \underset{1,\ldots ,40}{\arg\max}\;q^{\left(m\right)}=w^{\prime }\\ 0 & o.w. \end{array}\right.$$

Then, aggregating across all MCMC iterations, evaluate the proportion of MCMC samples in which each writer is most likely to be the true writer of the questioned document by

(10) $$\bar{p}=\frac{1}{M}\left[\sum_{m=1}^{M}v_{1}^{\left(m\right)},,,\sum_{m=1}^{M}v_{2}^{\left(m\right)},,,\sum_{m=1}^{M}v_{3}^{\left(m\right)},,,\ldots ,,,\sum_{m=1}^{M}v_{40}^{\left(m\right)}\right],$$

where $\sum_{i=1}^{40}{\bar{p}}_{i}=1$. Given that we restrict consideration to the closed‐set of writers, the $\bar{p}$ vector indicates the posterior probability of writership for each writer after accounting for variability in the parameters ***π***_*w*_ through the MCMC samples. In addition to the average posterior probability vector $\bar{p}$, we also determine the 2.5 and 97.5 percentile probability vectors to provide variability estimates.

**FIGURE 12:**
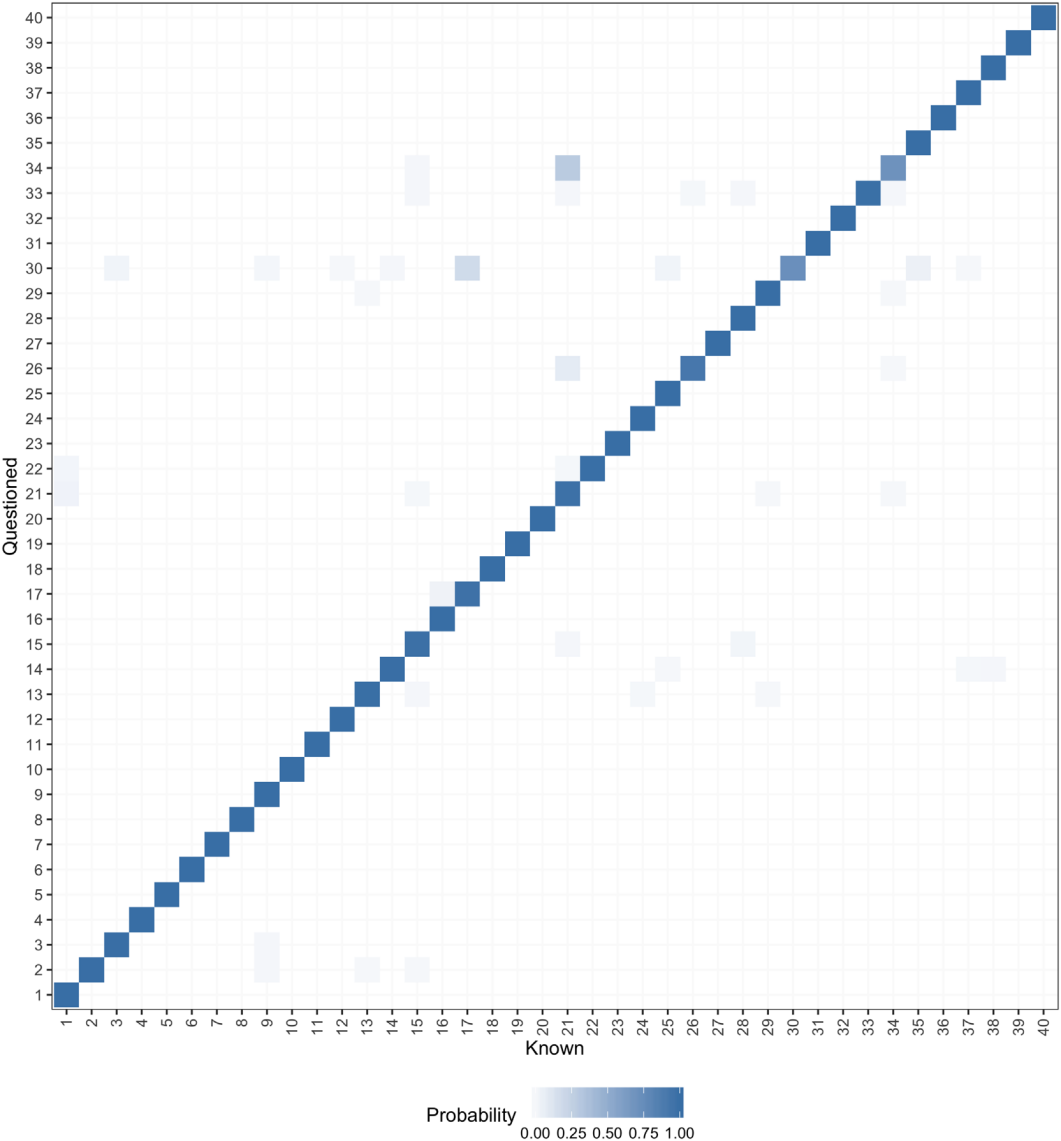
Writer identification results using the *K* = 40 clustering template to generate multinomial response vectors. Posterior probability of writership as defined in Equations (7), (8), (9), (10)) for each of the 40 holdout documents, one row for each. True writers of each questioned document are labeled on the left‐hand side. Columns are labeled by known writers in the closed training set. Cells are colored by elements of the $\bar{p}$ vectors for each holdout document. Thus, each row sums to one and can stand alone as a writership analysis of a single questioned document

The posterior predictive analysis defined in Equations (7), (8), (9), (10)) is repeated for each of the 40 questioned documents that were not used to fit the model. Of course, we know the true writer of each document. Thus, we can evaluate the predictive performance of the method by considering the amount of posterior probability $\bar{p}$ that was assigned to the correct writer of each questioned document. Figure 12 provides a graphical summary of these results. Each row presents the summary of the probabilities assigned to each of the 40 possible writers for a given questioned document. The diagonal, where the known writer of the questioned document matches the training set writer, indicates correct identification. When the number of clusters was fixed at *K* = 40, 98.06. % (95.0, 100.0) of all probability assignment goes to the true writer of the questioned document. The same procedure was followed using multinomial response vectors determined by the templates generated with *K* = 30, 50, and 60. Results are similar, and quantities of interest are provided in Table 1.

**TABLE 1:** Summaries of accuracy for the questioned document evaluations when each of the listed feature extraction techniques are used to create the multinomial response vector for a document

Feature extraction tool for creating groups		True writer $\bar{p}$ assignment (%)	(95% credible interval)
Clustering template	*K* = 30	97.48	(92.5, 100.0)
	*K* = 40	98.06	(95.0, 100.0)
	*K* = 50	96.96	(95.0, 97.5)
	*K* = 60	97.49	(92.5, 100.0)
Adjacency matrix		85.53	(77.5, 92.5)

The adjacency matrix groupings of Section 2.3 are used in the same modeling structure. The rigid nature of these groupings is such that approximately 60% of training graphs fall into the two most popular of 1764 adjacency groups. This pile‐up renders some of the graph group assignment information less valuable, since it tends to happen for most writers. In addition, many of the least populated groups appear only one or two times in the entirety of the training dataset. These groups do not contribute much reliable information to the multinomial data vectors which aim to capture general rates of group occurrence for a writer. In order to make the deterministic grouping results comparable to that of cluster grouping, we take the sparsely populated adjacency groups and collapse them into two new groups, making 40 total adjacency groups. We call the two new groups “moderate” and “rare” to describe their membership populations.

**FIGURE 13:**
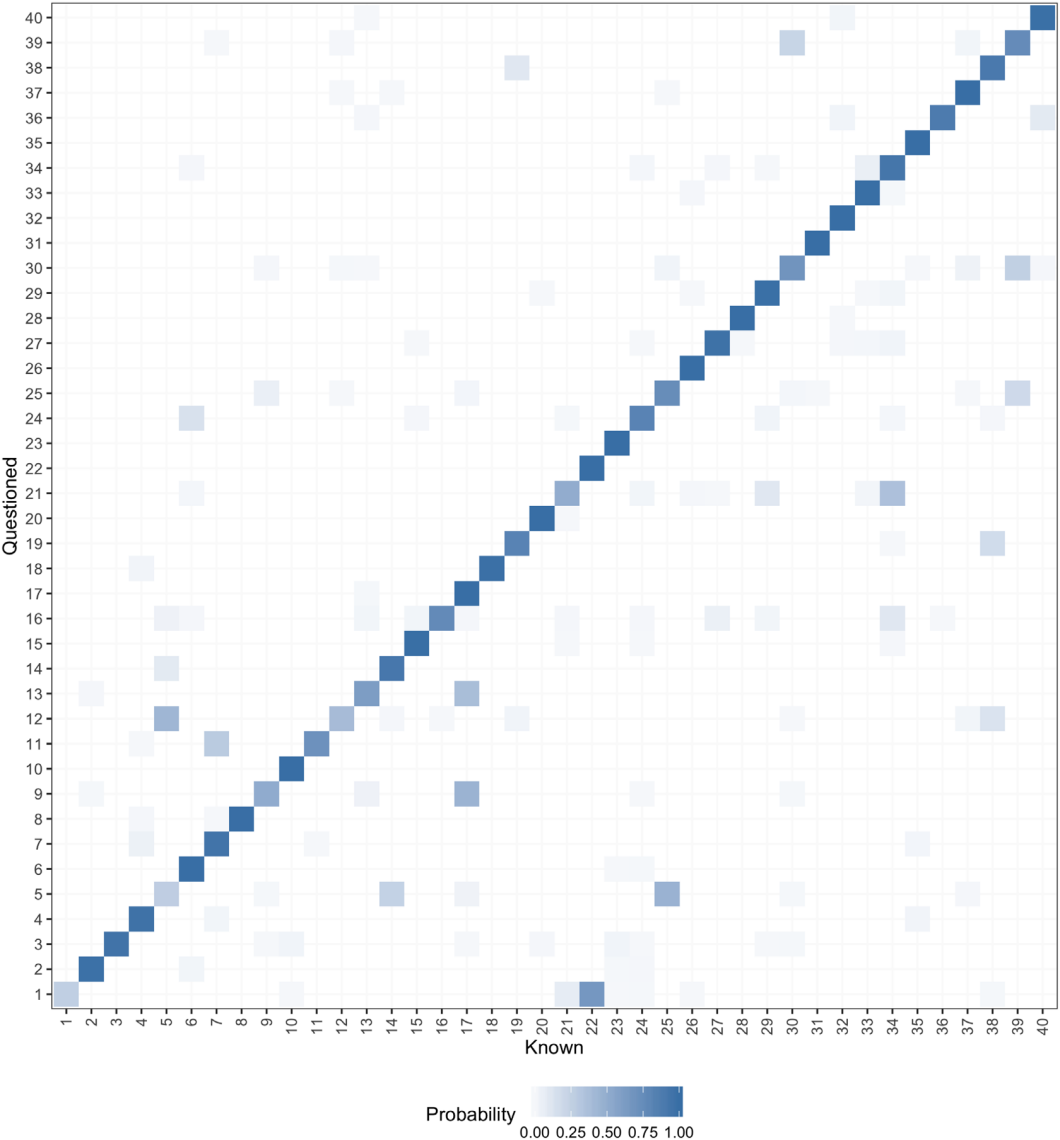
Writer identification results using the adjacency grouping method to generate multinomial response vectors. Posterior probability of writership as defined in Equations (7), (8), (9), (10)) for each of the 40 holdout documents, one row for each. True writers of each questioned document are labeled on the left‐hand side. Columns are labeled by known writers in the closed training set. Cells are colored by elements of the $\bar{p}$ vectors for each holdout document. Thus, each row sums to one and can stand alone as a writership analysis of a single questioned document

Figure 13 is similar to Figure 12, but the modeling results are products of the adjacency grouping method described in Section 2.3. Compared to the cluster grouping results of Figure 12, it is clear that there is more posterior probability assigned to the wrong writers (off‐diagonal) when the adjacency matrix based grouping is used. Quantities of interest that summarize the accuracy of the predictive goal using this grouping are included in Table 1.

## 5 DISCUSSION

In this paper, we propose a clustering algorithm for grouping graphs or small pieces of handwriting, and use an individual's propensity for creating graphs that belong to particular clusters to identify the writer of documents with unknown origin. Development of the clustering algorithm hinges on defining measures of distance and center for handwritten graphs. Our graph distance measure emulates a graph edit distance measure while leveraging additional attributes that the graphs possess. To illustrate the method, we use a subset of the CVL [8] handwriting database. With the CVL handwriting dataset we create clusters for graphs derived from handwriting samples and show that writers can be characterized and identified by examining the frequency with which they emit graphs to the various clusters. Cluster assignments for each graph in a document, based on a clustering template, serve as observations for a multinomial‐Dirichlet hierarchical model, and posterior prediction of writership for a set of questioned documents is discussed. We compare these identification results to those obtained using deterministic grouping of the graphs, showing that our cluster assignments improve upon the easily available, but volatile, deterministic assignments and proffers an improvement in model results.

In addition to the clustering algorithm, we describe the procedure by which a document is processed and converted into data. The handwriter R package reads, processes, and parses a document into graphs, the observations with which we work in the modeling steps. This processing takes an extremely complex data source—handwriting—and makes usable information readily available in the form of attributed graphs. The clustering and writership analysis as we did it would not be possible without this extraction.

Handwriting tends to be variable, both within and between writers, so when trying to cluster graphical structures that represent handwriting, we can observe surprising results. In particular, the mean calculation for diverse clusters results in non‐intuitive centers for the clusters. When clusters are more heterogeneous, the mean graph tends to shrink towards the centroid of the observations, and the order in which the observations are introduced to the weighted mean calculation can have small yet meaningful impacts on the resulting measure of center. This issue is mitigated in this paper by using exemplars rather than the raw weighted mean as the measure of center for the *K*‐means algorithm, but finding a way to calculate a stable mean for any cluster type could improve and simplify our algorithm.

Every distance measure tends to favor certain properties over others when assessing similarity, and the one we propose in this paper is no exception. There are many possible alternatives, and implementing a distance measure that prioritizes other features of handwriting is possible. For example, distance measures that do not take edge shape into account would prioritize edge location more heavily. On the other hand, discounting the location of an edge within a graph would lead to a measure that cares only about how similar the edge shapes are and not at all about where the edges lie in space. The distance measure that we implement is used because it leans evenly on many types of distance without over emphasizing any specific aspects of the graphs.

The flexible, dynamic *K*‐means clustering approach that we propose here results in groupings of handwritten graphical structures that appear to be discriminating and that may be useful in forensic practice. This said, the overall clustering method is applicable in any other field where the data come in the form of graphical objects, with nodes and edges.

## Data Availability

The data that support the findings of this study are openly available in Zenodo at https://doi.org/10.5281/zenodo.1492267, reference number 8.
